# PET imaging of hepatocellular carcinoma with anti-1-amino-3-[^18^F]fluorocyclobutanecarboxylic acid in comparison with l-[*S*-methyl-^11^C]methionine

**DOI:** 10.1186/s13550-019-0519-4

**Published:** 2019-05-22

**Authors:** Olga Sergeeva, Yifan Zhang, Jonathan D. Kenyon, Galen A. Miller-Atkins, Chunying Wu, Renuka Iyer, Sandra Sexton, Patrick Wojtylak, Amad Awadallah, Wei Xin, E. Ricky Chan, James K. O’Donnel, Zhenghong Lee

**Affiliations:** 1 Radiology, Case Western Reserve University, Cleveland, OH USA; 2 Biology, Case Western Reserve University, Cleveland, OH USA; 3 Institute for Computational Biology, Cleveland, OH USA; 4 Medical Oncology, Rowell Park Cancer Institute, Buffalo, NY USA; 5 Nuclear Medicine, Radiology, University Hospitals Cleveland Medical Center, Cleveland, OH USA; 6 Pathology, University Hospitals Cleveland Medical Center, Cleveland, OH USA

**Keywords:** Amino acid transporter, Hepatocellular carcinoma, Woodchuck model, PET imaging

## Abstract

**Purpose:**

[^11^C]methionine ([^11^C]Met) was used for cancer imaging based on upregulated amino acid transport and protein synthesis in different tumor types. However, the short half-life of ^11^C decay limited further clinical development of [^11^C]Met. Synthetic amino acid analog anti-1-amino-3-[^18^F]fluoro-cyclobutyl-1-carboxylic acid ([^18^F]FCABC) was developed and FDA-approved for PET imaging of recurrent prostate cancer. This study investigated “repurposed” [^18^F]FACBC for PET imaging of primary liver cancer such as hepatocellular carcinoma (HCC) in comparison with [^11^C]Met.

**Methods:**

[^11^C]Met was synthesized in the lab, and [^18^F]FACBC was purchased from a commercial outlet. A clinically relevant animal model of spontaneously developed HCC in the woodchucks was used for PET imaging. Bioinformatics analysis was performed for the expression of amino acid transporters responsible for radiotracer uptake and validated by PCR. Dynamic PET scans of [^11^C]Met and [^18^F]FACBC were acquired within 1 week. Standardized uptake value (SUV) was calculated for regions of interest (ROIs) defined over HCC and a liver background region. H&E staining and immunohistochemical (IHC) staining were performed with harvested tissues post-imaging.

**Results:**

Higher expression of ACST2 and LAT1 was found in HCC than in the surrounding liver tissues. PCR validated this differential expression. [^11^C]Met and [^18^F]FACBC displayed some differences in their uptake and retention in HCC. Both peaked in HCC with an SUV of 3.5 after 10 min post-injection. Met maintained a plateaued contrast uptake in HCC to that in the liver while [^18^F]FCABC declined in HCC and liver after peak uptake. The pathological assessment revealed the liver tumor as moderately differentiated similar to the human HCC and proliferative.

**Conclusion:**

Both [^18^F]FACBC and [^11^C]Met showed uptake in HCC through the use of a clinically relevant animal model of woodchuck HCC. The uptake and retention of [^18^F]FACBC and [^11^C]Met depend on their metabolism and also rely on the distribution of their principal amino acid transporters.

## Background

**Fig. 1:**
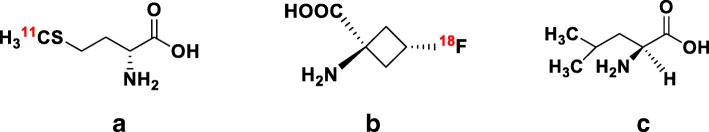
Structures of amino acids. From left, **a** l-[*S*-^11^C]methionine, **b** [^18^F]FACBC, and **c** l-leucine

Currently, there is no “good” radiotracer for PET imaging to supplement the standard radiological imaging of primary liver cancers such as hepatocellular carcinoma (HCC), the third leading cause of cancer-related death world-wide [1]. Natural amino acids such as methionine (Met) was radiolabeled with ^11^C for cancer imaging based on upregulated amino acid transport and protein synthesis in different tumor types, which resulted in a high uptake of Met seen with PET imaging [2]. The most notable results so far came from brain tumor studies where lower background uptake of Met in the brain led to more favorable tumor-to-background uptake ratios than could be achieved with a commonly used glucose analog, 2-[^18^F]fluoro-2-deoxy-d-glucose (FDG) [3]. Uptake of FDG reflects the increased cellular glucose metabolism, which has dramatically improved patient management in a large variety of cancers. Unfortunately, FDG has severe limitations for imaging primary liver cancers such as HCC due to a high false negative rate as many HCCs have relatively low FDG uptake [4]. A different radiotracer is needed for effective PET imaging of liver cancer.

l-[*S*-*methyl*-^11^C]methionine ([^11^C]Met, Fig. 1a) was tested for HCC imaging [5, 6]. Although the initial clinical study showed a high uptake of [^11^C] Met in HCC [6, 7], the 20-min short half-life of ^11^C decay limited further clinical development of [^11^C]Met for liver cancer applications. The non-natural amino acids provided an alternative. The advantages of these synthetic amino acid analogs include their ability to incorporate longer-lived radionuclides such as ^18^F, which makes routine clinical applications feasible through commercial batch production for regional distribution, and the lack of radiolabeled metabolite formation, which simplifies kinetic analysis and avoids possible confounding accumulation of activity in non-target tissues.

**Fig. 2:**
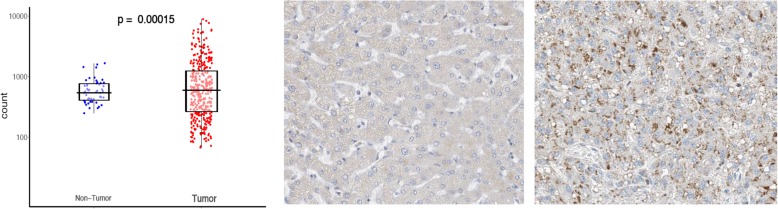
ASCT2 expression in human HCC. The left panel: RNA-seq (TCGA) showing differential expression of ASCT2 between HCC and liver tissues. Staining of the liver (middle panel) and HCC (right panel) with antibody HPA035239 against ASCT2 (Human Protein Atlas)

Synthetic amino acid analog *anti*-1-amino-3-[^18^F]fluoro-cyclobutyl-1-carboxylic acid (*anti*-[^18^F] FACBC, short for [^18^F] FACBC Fig. 1b) was developed and FDA-approved as Fluciclovine (^18^F) or Axumin® for PET imaging of recurrent prostate cancer. This study was designed to investigate [^18^F]FACBC when “repurposed” for PET imaging of primary liver cancer such as HCC in comparison with [^11^C]Met. The mechanism of [^11^C]Met uptake in HCC was elucidated through our previous investigations [8, 9]. Contrariwise, [^18^F]FACBC is not metabolized by design, and its uptake is mediated mainly by amino acid transporters, which include l-type amino acid transporter 1 (LAT1), LAT2, and also the *a*lanine, *s*erine, and *c*ysteine preferring transport system 2 (ASCT2), all of which are highly conserved among the species [10]. The affinity of these amino acid transporters for [^18^F]FACBC is well characterized [11] in the order of high to low (Michaelis-Menten kinetics, *K*_*m*_, in micromolar) as ASCT2 (*92.0 ± 32.0*) >> SNAT2 (*222.0 ± 29.3*) ≥ LAT1 (*230.0 ± 84.5*) >> LAT2 (*738.5 ± 87.6*), where SNAT2 is the Na^+^-coupled neutral amino acid transporter 2 (also known as solute carrier family 38 member 2, or SLC38A2) while ASCT2, LAT1, and LAT2 are likewise known as SLC1A5, SLC7A5, and SLC7A8, respectively. Our analyses (Fig. 2) of the human RNA-seq data from The Cancer Genome Atlas (TCGA) showed a higher level of expression of ACST2 in HCC than in the surrounding liver tissues and LAT1, which revealed a similar difference. In comparison, SNAT2 and LAT2 showed no differential expression between liver tumor and tissue. ASCT2 seemed to be the main active transporter that is responsible for [^18^F]FACBC uptake in HCC. We thus tested [^18^F]FACBC for PET imaging of HCC, in comparison with [^11^C]Met, by using a unique animal model of HCC with clinical relevance.

As a clinically relevant animal model, the eastern woodchuck (*Marmota monax*) developed HCC after chronic viral hepatitis infection when it harbors a DNA virus—the woodchuck hepatitis virus (WHV), a member of the family Hepadnaviridae, of which human hepatitis B virus (HBV) is the prototype. Like HBV, WHV infects woodchuck liver to cause acute and chronic hepatitis, which leads to the development of HCC within 2–4 years of life. The HCC in woodchucks is considered as recapitulating the human HCC with similar pathology and natural history [12–14]. Our bioinformatics analysis (see the “Results” section) showed that ASCT2 and LAT1 have high expression in HCC than in the surrounding liver tissues in the woodchuck models. In particular, the base mean of ASCT2 expression is higher than that of LAT1 in HCC, which might have led to different uptake patterns between [^11^C]Met and [^18^F]FACBC during PET imaging.

## Materials and methods

### Animal models

Three woodchucks weighted 3.6–4.8 kg (*n* = 3, averaged 4.0 kg) and aged 2–3 years old were ultrasound-imaged at Roswell Park Cancer Institute (Buffalo, NY) and selected for shipment to Case Western Reserve University (Cleveland, OH) when the liver nodules were larger than 20 mm in size. A venous access port (SAI Infusion Technologies; Elgin, IL) was surgically implanted in each animal to facilitate radiotracer injections for all PET scans. The port was flushed regularly with heparinized saline. The food was taken away 4–5 h before each PET imaging session while drinking water was always kept. All procedures are approved by the Institutional Animal Care and Use Committee of the University (Protocol #2014-0085).

### Bioinformatics

TCGA data were downloaded from the public TCGA Liver Hepatocellular Carcinoma database (TCGA-LIHC). The data include a total of 371 RNA-seq gene expression results collected from both tumor and non-tumor human tissues. The TCGA-LIHC data were all sequenced using Illumina platforms and available as “raw” (i.e., un-normalized) read counts for each gene for each sample. Data were then reformatted and sent through the DESeq2 workflow in R [15] including a pre-filtering step, where genes with very low gene counts are excluded, a normalization step to aid in comparing genes across samples, calculating the log_2_ fold change between non-tumor and tumor samples for each gene, and the differential expression between non-tumor and tumor genes with a Wald test. The final results yield the base mean for each gene, the log_2_ fold change, the standard error for the fold change, the Wald test statistic, and the raw and adjusted *p* value for multiple testing correction using FDR.

The woodchuck data were collected from the NCBI Gene Expression Omnibus (accession number GSE36545 and BioProject PRJNA155585). The data include 102 samples (GSM896624-GSM896725) from 13 woodchucks with a total of 42 tumor samples and 60 non-tumor samples [16]. The data obtained was from a custom NimbleGen Woodchuck Gene Expression HX3 Microarray. The downloaded data are normalized gene expression data for each sample, formatted in parallel to the TCGA dataset although the two sets of data were processed differently due to the difference in the dynamic range inherent to each technology. Nine outlying samples were identified with principal component analysis and subsequently excluded from the analysis. Log_2_ fold change and *t* tests were then calculated to compare the gene expression between non-tumor and tumor samples. All resulting *p* values were FDR corrected to adjust for multiple testing.

Levels of gene expression of amino acid transporters, ASCT2, LAT1, SNAT2, and LAT2, between liver tumors and non-tumor liver tissues, were tallied from the databases. The human data came from TCGA, and the woodchuck data from the customized microarray. The homology of amino acid sequences between human ([*Homo sapiens*]) and woodchuck ([marmot]) proteins (ASCT2 and LAT1) was determined by using Protein Basic Local Alignment Search Tool (BLAST) (https://blast.ncbi.nlm.nih.gov/Blast.cgi). Specifically, the homology between the two species for solute carrier family 1 member 5 isoform 1 (ASCT2) (GenBank: ACX53626.1 [*Homo sapiens*] and NCBI Reference Sequence: XP_015352142.1 [*Marmota marmota* marmota]) was searched. Similarly, the homology between the two species for Na^+^-independent neutral amino acid transporter (LAT1) (GenBank: BAB70708.1 [*Homo sapiens*] and NCBI Reference Sequence: XP_015351465.1 [*Marmota marmota* marmota]) was also searched using BLAST.

### Radiotracers

[^11^C]Met was synthesized based on the solid phase [^11^C] methylation of the precursor l-homocysteine thiolactone on a C-18 Sep-Pak. Briefly, the cyclotron-derived [^11^C] carbon dioxide was converted to [^11^C]methyl iodide ([^11^C]CH_3_I) in an automatic synthesis module Tracerlab FXc (General Electric Medical Systems). The online formed [^11^C]CH_3_I was transferred under stream of helium (20 mL/min) onto a C-18 Sep-Pak Plus, which was previously loaded with 210 μL of a solution of 7.7 mg l-homocysteine thiolactone hydrochloride dissolved in 500 μL of 0.5 M NaOH in ethanol/water 50/50. After 5 min, [^11^C]Met was eluted with 2.5 ml of PBS (50 mM) and was purified by semi-preparative HPLC (C-18 column, NaH_2_PO_4_•2H_2_O (50 mM) in 2% ethanol as mobile phase). The final product was formulated with the same phosphate buffer and saline. The final volume was 10 mL, which was passed through a 0.22 mm filter. The radiochemical yield was 55%, and radiochemical purity was greater than 98% determined by RP-HPLC with Nucleosil C18 column (3 × 250 mm) using NaH_2_PO_4_•2H_2_O (50 mM) with 2% ethanol at the flow of 0.3 ml/min.

Clinical doses of [^18^F]FACBC (specific activity 580–820 MBq/μmol, radiochemical purity 99%) was purchased from Blue Earth Diagnostics (BED, Burlington, MA) and delivered by the local outlet of PETNET (onsite at University Hospitals Cleveland Medical Center, Cleveland, OH), BED’s production partner.

### PET imaging and data analysis

The spontaneously developed HCC in the woodchucks was used for PET imaging experiments with the clinical Ingenuity PET/CT scanner (Philips, Cleveland, OH). Each animal was placed prone in the clinical PET/CT scanner and under 3% isoflurane gas anesthesia. After a low-dose CT scan, [^18^F]FACBC was injected intravenously (i.v.) through the implanted venous access port and followed by a dynamic PET scan of 60 min in list mode on the clinical PET/CT system as the woodchucks with an average weight of 3.5 kg did not fit into the microPET. Briefly, the dynamic scan was acquired upon injection of 37~56 MBq (1.0~1.5 mCi) of [^18^F]FACBC i.v. into the woodchuck. PET acquisition was re-binned into a total of 21 frames: 10 × 30 s, 5 × 1-min, 2 × 5-min frames, and 4 × 10 min, respectively. CT-based attenuation correction was embedded in the iterative reconstruction of PET images as part of the standard package supplied by the vendor. Another dynamic PET scan with [^11^C]Met was performed identically on the same animal within 1 week of the [^18^F]FACBC scan.

Standardized uptake value (SUV) [17] was calculated for regions of interest (ROIs) defined over focal uptakes of [^18^F]FACBC or [^11^C]Met as well as a nearby ROI for liver background away from focal uptakes, similar to that for FDG uptake [18], and time-activity curves in the unit of SUV were generated for these ROIs for each radiotracer. The uptake ratio between tumor and liver background was also generated for each tracer.

### qRT-PCR

The primers for qRT-PCR were designed at The Custom TaqMan® Assay Design Tool based on marmot mRNA sequences for required genes: ASCT2 (SLC1A, custom TaqMan gene expression assay AP9HKRJ based on XM_015496656.1 mRNA sequence), LAT1 (SLC7A5, assay AP7DR6M based on XM_015495979.1 mRNA sequence), and endogenous control gene GAPDH (assay AP2W9P7 based on XM_015500718.1 mRNA sequence). RNA was extracted from tissue using Qiagen miRNeasy Mini Kit (Cat. No. 217004, Qiagen) according to the manufacturer’s instructions. Total RNA (0.1 μg for each reaction) was used to generate complementary DNA (cDNA) with the High-Capacity RNA-to-cDNA Kit (Cat. No. 4387406; Applied Biosystems). qRT-PCR was performed on a StepOne Plus real-time thermocycler with 1.33 mL of cDNA for each reaction and the TaqMan Universal Master Mix II, with UNG (Cat. No. 4440038; Applied Biosystems). Expression data was obtained for each gene from each sample as threshold cycle (Ct). ΔCt was calculated as the Ct of the endogenous control gene minus the Ct of the gene of interest. ΔΔCt was then calculated as the ΔCt of the reference sample minus the ΔCt of another sample. This sets the ΔΔCt of the reference sample to 0. The relative quantification of gene expression (RQ) was calculated as 2^−(ΔΔCt)^. This yields an RQ for the reference sample as 1. Samples with more transcripts than the reference sample will have negative ΔΔCt scores and larger RQ values.

### Histology analysis

After PET imaging sessions, the animals were euthanized with liver tissues harvested. Some of the tissues were fresh-frozen and later used for PCR described above. Other tissues were fixed in formalin for H&E staining as well as immunohistochemical (IHC) staining for proliferative status using the anti-PCNA antibody [19] (PC10, from Abcam). The liver pathologist evaluated the liver tumor based on the H&E staining.

## Results

### Bioinformatics

Analysis of the RNA-seq data from TCGA revealed a wide range in the expression of amino acid transporters ASCT2 and LAT1 in both human HCC and the surrounding liver parenchyma. Figure 2 showed that the base main count of ASCT2 in HCC, *1187.010*, is significantly higher (adjusted *p* = 0.00015) than that in the surrounding liver tissues, *636.117*; while in a similar comparison, *1051.528* vs. *861.018*, is not (adjusted *p* = 0.21055) for LAT1. The other two amino acid transporters SNAT2 and LAT2 did not show differential expression between HCC and liver tissues. Selective histology from TCGA-related protein atlas displayed differential staining of ASCT2 proteins between human HCC and liver tissues (Fig. 2 mid- and right panels).

**Fig. 3:**
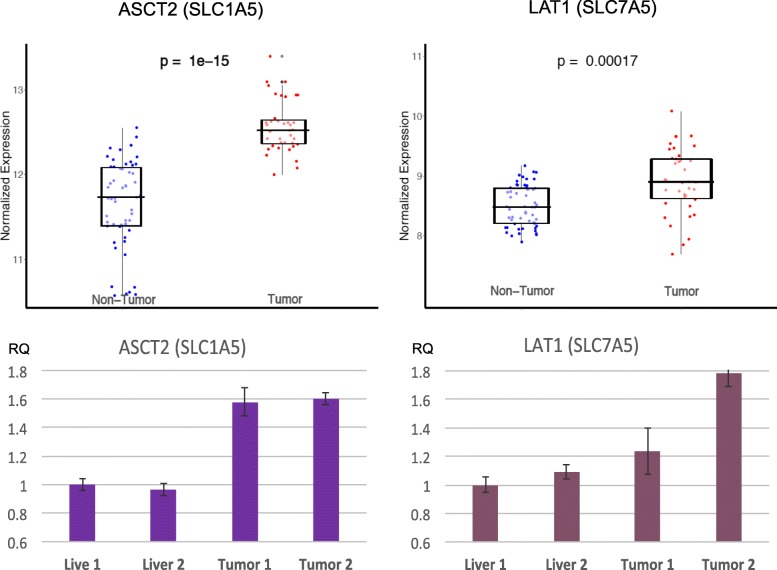
l-amino acid transporters. Top: Expression data (microarray) of ASCT2 (left) and LAT1 (right) between HCC and the surrounding liver tissues from the woodchucks. Bottom: qRT-PCR data of ASCT2 (left) and LAT1 (right) of sampled liver and tumor tissues

Analysis of the customized microarray data from using the woodchuck samples showed a similar wide range in the expression of the transporters in woodchuck HCC and the surrounding woodchuck hepatic tissues (Fig. 3, upper panels). The level of transcripts of both ASCT2 and LAT1 are significantly higher in the woodchuck HCC than in the surrounding hepatic tissues. The base means for ASCT2 were *12.564* vs. *11.663* for tumor vs. liver tissues, respectively. Those for LAT1 were *8.918* vs. *8.495*, accordingly. The results of qRT-PCR (Fig. 3, lower panels) from using the woodchuck primers validated the differential expression levels of both ASCT2 and LAT1 between HCC and the surroundings.

**Fig. 4:**
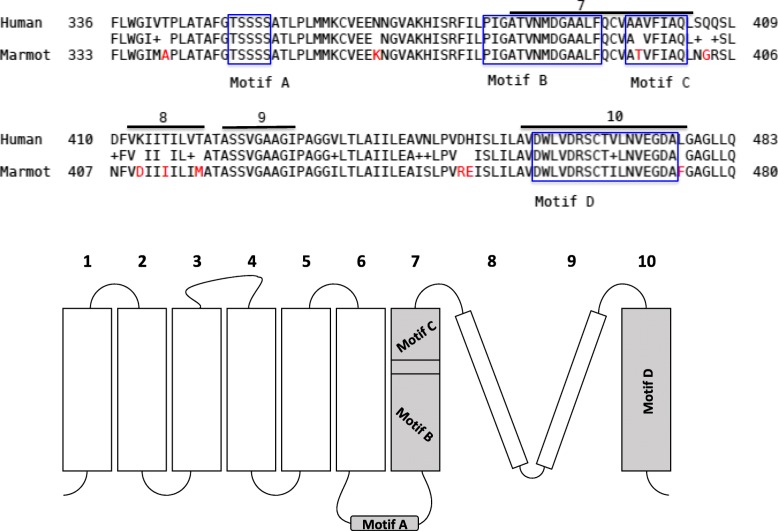
Top: BLAST of protein sequence between human and woodchuck (marmot) ASCT2. The upper bars highlight the trans-membrane segments, and the blue boxed are the key motifs involved in amino acid transport. The red letters represent the differences between the two species in amino acid residues. Bottom: membrane model of ASCT2 towards the C-terminal. While segments 7 and 10 are helices, 8 and 9 form a re-entrant loop-pore structure with conserved motifs (A, B, C, and D), adapted from Ref. [10]

Using amino acid sequence BLAST, the homology between human (*Homo sapiens*) and woodchuck (marmot) proteins was identified. This search revealed homology between the two species for ASCT2 and LAT1, 78% and 92% identity, respectively. BLAST results between the two species for both ASCT2 and LAT1 are appended in the supplementary. ASCT2 exhibited a slightly lower homology between the two species for the entire sequence of the protein. Yet Fig. 4 displays the high similarity in a segment that is key to substrate critical amino acid sequences between human and marmot in ASCT2. Specifically, the C-terminal portion exhibits a higher degree of identity than the whole sequence. An illustrated membrane model, in which a re-entrant loop-pore structure contains highly conserved motifs, is accompanied in Fig. 4 for localizing the segment.

### PET imaging

**Fig. 5:**
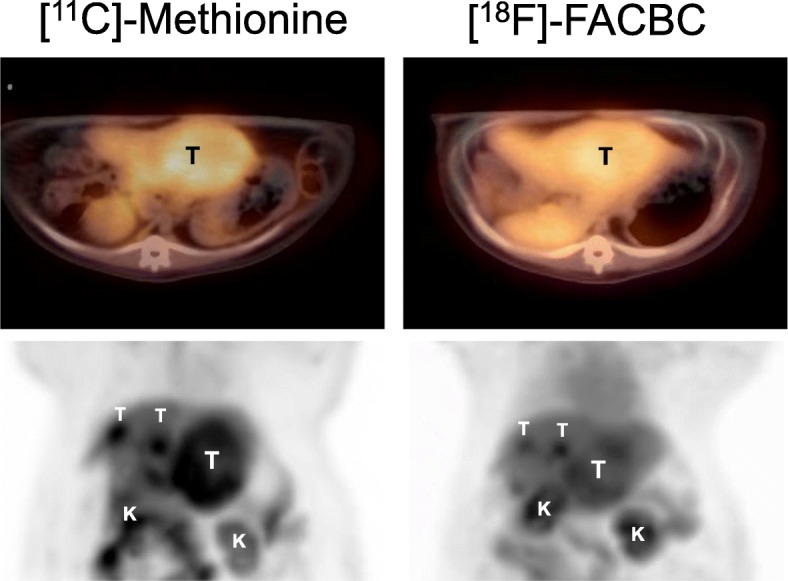
Comparison between [^11^C]Met and [^18^F]FACBC for PET imaging of HCC taken between 15 and 20 min post-injection. Top: PET/CT overlay (axial view). Bottom: maximum intensity projection (MIP, coronal view). T tumor (the large tumor in one lobe, also shown on the axial view, and two smaller tumors in a lobe behind); K kidney

**Fig. 6:**
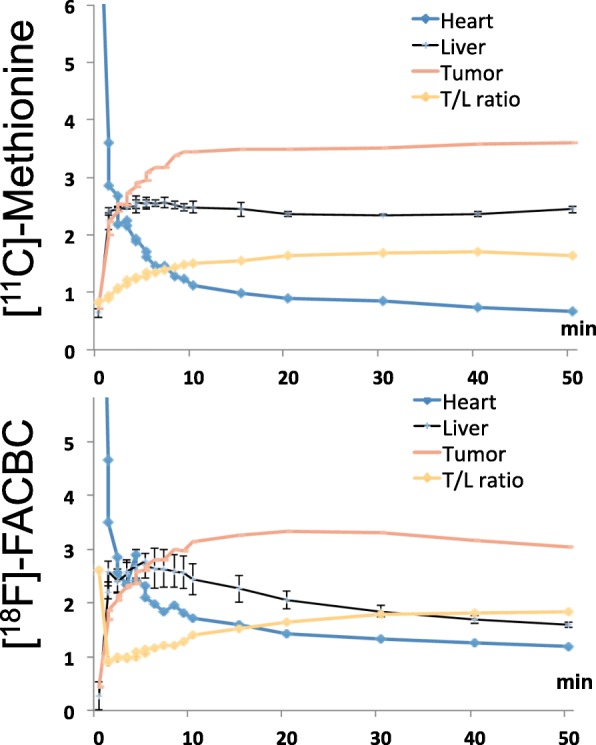
Regional time activity curves (in SUV) generated from the dynamic PET scans of the same animal with [^11^C]Met and [^18^F]FACBC, respectively, along with their tumor to liver background (T/L) ratios

Figure 5 showed the comparison of PET imaging with woodchuck models of HCC using [^11^C]Met and [^18^F]FACBC for the uptake at 15–20 min post tracer injection. PET/CT overlay of the axial views across the liver showed uptake of both radiotracers in HCC and the coronal views displayed in maximum intensity projection (MIP) showed, beside HCC, uptake in the kidney, spleen, and lower abdomen, for which [^11^C]Met has a stronger intensity. Different uptake patterns were observed from using different radiotracers on the same animal model. Regional time activity curves (in the unit of SUV, Fig. 6) were generated from the dynamic PET scans with [^11^C]Met and [^18^F]FACBC, respectively, along with the uptake ratio between the tumor and liver background for each. By 10 min post-injection when the uptake of both [^11^C]Met and [^18^F]FACBC in HCC peaked at SUV = 3.5 and 3.3, respectively. When cleared out of circulation (heart), the uptake of [^11^C]Met almost plateaued in both HCC and the liver for the remaining of the dynamic scan with a tumor to liver background (T/L) ratio maintained around 1.6~1.7 while that of [^18^F]FACBC started to decrease slowly in HCC as well as in the liver tissues along the time, but with a T/L ratio increasing to 1.83.

### Histology

**Fig. 7:**
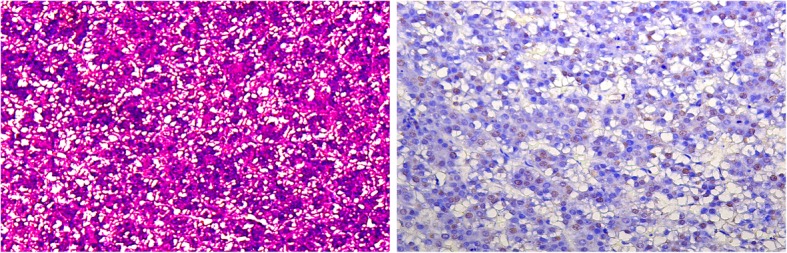
H&E staining (10×, left) displayed moderately differentiated HCC in the woodchuck, and PCNA staining (20×, right) showed an intermediate level of nuclear (brown) staining indicating an ample degree of proliferation in the liver tumor

Figure 7 showed H&E staining (10×) and PCNA staining (20×) in HCC. The tumors were assessed as mostly moderately differentiated with a noticeable level of inflammation. PCNA (nuclear) staining was obvious indicating the proliferative nature of the tumor with radiotracer uptake.

## Discussion

Radiolabeled amino acids were developed to image the increased level of amino acid transport that occurs in many tumor cells in contrast to the surrounding tissues [20]. Naturally occurring amino acids are usually labeled with ^11^C such as [^11^C]Met, which limits their use in routine clinical scans. The advantages of these non-natural, non-metabolized amino acid analogs include the ability to incorporate longer-lived radionuclides such as ^18^F and the lack of radio-metabolite formation.

Synthetic amino acid analogs with the ^18^F label are preferable for clinical cancer imaging if they achieve a comparable tumor-to-background uptake ratio to that of natural amino acids. (*4S*)-4-(3-^18^F-fluoropropyl)-l-glutamine ([^18^F]FSPG) is an l-glutamate derivative that is a substrate to the sodium-dependent cystine-glutamate exchange-transporter ×(C)(−) [21]. ^18^F-FSPG was tested in a human trial and showed contrast uptake in moderately and poorly differentiated HCCs, but remained challenging with well-differentiated HCC, similar to FDG [22]. There is a radiolabeled glutamine, l-[5-^11^C] glutamine, and analog ^18^F-(*2S*,*4R*)4-fluoroglutamine, that were designed for imaging glutaminolysis in tumors [23, 24]. However, both displayed a high liver background uptake, therefore are not suitable for liver cancer imaging. The same is true for l-[2-^18^F]fluorotyrosine [25], which was initially developed for imaging increased activity of amino acid transporters in brain tumors.

There are other small molecule radio-ligands tested for PET imaging of HCC. Among them, [^11^C]acetate and [^11^C]choline (and [^18^F]fluoridated choline) have shown uptake in HCC [26–29], but their utility needs to be clinically tested. Recently, Ga-68-labeled short peptide ligand against prostate-specific membrane antigen (PSMA), developed originally for PET imaging of prostate cancer, was tested for PET imaging of HCC [30, 31]. Since the PSMA targets seemed to be on tumor-associated vascular endothelial cells, not on liver cancer cells [32], further investigations are needed.

In this study, [^18^F]FACBC was tested in comparison with [^11^C]Met for PET imaging of HCC using a clinically relevant animal model of spontaneous HCC. [^18^F]FACBC was developed and approved for PET imaging of prostate cancer. For prostate cancer imaging, Na^+^-coupled neutral amino acid transporter SNAT2 and another neutral amino acid transporter ASCT2 as well as Na^+^-independent l-amino acid transporter LAT1 are all upregulated. A recent independent clinical PET imaging study [33] discovered [^18^F]FACBC uptake in human HCC by accident. While ASCT2 is the main amino acid transporter that is responsible for [^18^F]FACBC uptake [34], LAT1 is the main transporter responsible for [^11^C]Met uptake [35].

ASCT2 and LAT1 are upregulated in HCC based on bioinformatics analysis and validated by PCR (Figs. 2 and 3), and both [^11^C]Met and [^18^F]FACBC displayed uptake in liver cancer. For the woodchuck models of HCC, which is a spontaneously developed HCC after chronic hepatitis infection, no human viruses or tissues are involved. The pathological assessment revealed the liver tumor as moderately differentiated similar to the human HCC that is proliferative in nature (Fig. 7). The woodchuck indigenous amino acid transporters exhibit a high degree of homology in amino acid sequence to the human orthologs (Fig. 4 and supplementary). This homology most likely allowed the radiolabeled amino acids, [^11^C]Met and [^18^F]FACBC, developed as substrates for human transporters to be transported also by the woodchuck’s indigenous transporters during PET imaging.

Comparison between [^11^C]Met and [^18^F]FACBC for their uptake and retention in HCC revealed some differences between the two radiolabeled amino acids. The difference in uptake (Fig. 5) can be explained by the possibly not entirely overlaying expression or distribution pattern of the two amino acid transporters, ASCT2 and LAT1, as each is responsible for a preferred radiotracer, [^18^F]FACBC and [^11^C]Met, respectively. Future work will focus on validation by co-IHC staining the two transporters to show the differential localization. The difference in retention/clearance, mainly in the liver as reflected by the T/L ratio (Fig. 6), can be explained by differential metabolism of the two radiotracers. Hepatic metabolism of Met was studied previously [8, 9]. Briefly, after transport by the high affinity and low capacity LAT1, the low affinity but high-capacity LAT2, and also ASCT2, Met is converted primarily into amino-acyl-tRNA by the enzyme amino-acyl RNA synthetase. This process is the first step in protein synthesis, which is enhanced in liver cancer as well as in tumors. [^11^C]Met can also be converted into *S*-adenosylmethionine via methionine adenosyltransferase and through PE-methylation pathway into phospholipid incorporation in the liver and liver cancer. This explains the apparent plateau (almost steady T/L ratio) in [^11^C]Met uptake in both HCC and liver parenchyma as tracer metabolism was on-going during PET imaging and the radio-metabolites were retained.

On the other hand, [^18^F]FACBC is not metabolized by design and would not be retained after transported. Thus, efflux of a non-metabolized could potentially explain the decline of the initial uptake of [^18^F]FACBC in HCC. Figure 6 indicates that while the [^18^F]FACBC signal in both liver and HCC decreased over time, the liver background signal fell faster, which resulted in an increase in T/L ratio, indicating increased intracellular to extracellular transport. Another l-amino acid transporter that could be responsible for this observation could be SLC43A1 since it is involved in the efflux of branched chain amino acids from the liver to blood [36, 37]. Shown in Fig. 1, [^18^F]FACBC is an analog of branched-chain amino acid l-leucine and a likely substrate for SLC43A1. TCGA data showed a high level of SLC43A1 expression in both liver and liver cancer with a much tighter range of expression in the liver than in HCC. Future work would also need to include co-IHC staining of SCL43A1 for liver and HCC in addition to those for ASCT2 and LAT1, as well as conducting efflux assays of [^18^F]FACBC with liver tissues. The low extracellular levels of competing amino acids may also contribute to the efflux of [^18^F]FACBC.

## Conclusion

[^18^F]FACBC is a radiopharmaceutical approved by FDA. “Repurposing” it for use other than prostate cancer will be straightforward. The neutral amino acid transporter (ASCT2) mainly responsible for [^18^F]FACBC uptake in the prostate cancer seemed also to be the primary transporter that is upregulated in HCC. Both [^18^F]FACBC and [^11^C]Met depicted HCCs as discovered through the use of a clinically relevant animal model of spontaneously developed HCC in the woodchucks. The uptakes of [^18^F]FACBC and [^11^C]Met rely on different principal amino acid transporters, ASCT2 mainly for [^18^F]FACBC and LAT1 for [^11^C]Met, which could lead to different uptake patterns of the two radiotracers for the same HCCs. Once further developed and validated, PET imaging with [^18^F]FACBC will potentially help a number of clinical management decisions.

## Abbreviations

ASCT: Alanine, serine, and cysteine preferring transport system
BLAST: Basic Local Alignment Search Tool
cDNA: Complementary DNA
FACBC: Anti-1-amino-3-[^18^F]fluoro-cyclobutyl-1-carboxylic acid
FDG: 2-[^18^F]fluoro-2-deoxy-d-glucose
FDR: False discovery rate
FSPG: (*4S*)-4-(3-^18^F-fluoropropyl)-l-glutamine
H&E: Haemotoxylin and eosin
HBV: Hepatitis B virus
HCC: Hepatocellular carcinoma
IHC: Immunohistochemistry
LAT: l-type amino acid transporter
Met: Methionine
MIP: Maximum intensity projection
NCBI: National Center for Biotechnology Information
PCNA: Proliferating cell nuclear antigen
qRT-PCR: Quantitative reverse transcription polymerase chain reaction
ROI: Region of interest
RQ: Relative quantification
SLC: Solute carrier
SNAT: Na^+^-coupled neutral amino acid transporter
SUV: Standardized uptake value
TCGA: The Cancer Genome Atlas
WHV: Woodchuck hepatitis virus
